# TMEM43 Protects against Sepsis-Induced Cardiac Injury via Inhibiting Ferroptosis in Mice

**DOI:** 10.3390/cells11192992

**Published:** 2022-09-26

**Authors:** Zhen Chen, Zhe Cao, Feng Gui, Mengli Zhang, Xian Wu, Huan Peng, Bo Yu, Wei Li, Fen Ai, Jun Zhang

**Affiliations:** 1 Department of Emergency, The Central Hospital of Wuhan, Tongji Medical College, Huazhong University of Science and Technology, Wuhan 430014, China; 2 Department of Cardiovascular, The Central Hospital of Wuhan, Tongji Medical College, Huazhong University of Science and Technology, Wuhan 430014, China

**Keywords:** sepsis-induced cardiac injury, transmembrane protein 43, ferroptosis, P53, ferrostatin-1

## Abstract

A previous study found that transmembrane protein 43 (TMEM43) was highly associated with arrhythmogenic right ventricular dysplasia/cardiomyopathy. However, as a transmembrane protein, TMEM43 may be involved in ferroptosis in cardiovascular disease. In this study, we aimed to explore the role of TMEM43 in lipopolysaccharide (LPS)-induced cardiac injury and the underlying mechanism. Mice were injected with LPS (10 mg/kg) for 12 h to generate experimental sepsis. Mice were also subjected to AAV9-shTMEM43 to knock down TMEM43 or AAV9-TMEM43 to overexpress TMEM43 in hearts. H9c2 rat cardiomyocytes were also transfected with Ad-TMEM43 or TMEM43 siRNA to overexpress/knock down TMEM43. As a result, TMEM43 knockdown in hearts deteriorated LPS-induced mouse cardiac injury and dysfunction. LPS increased cardiac ferroptosis as assessed by malonaldehyde (MDA) and cardiac iron density, which were aggravated by TMEM43 knockdown. Moreover, TMEM43 overexpression alleviated LPS-induced cardiac injury, dysfunction, and ferroptosis. In vitro experiments showed that TMEM43 overexpression inhibited LPS-induced lipid peroxidation and cardiomyocyte injury while TMEM43 knockdown aggravated LPS-induced ferroptosis and injury in cardiomyocytes. Mechanistically, LPS increased the expression of P53 and ferritin but decreased the level of Gpx4 and SLC7A11. TMEM43 could inhibit the level of P53 and ferritin enhanced the level of Gpx4 and SLC7A11. Furthermore, ferrostatin-1 (Fer-1), a specific inhibitor of ferroptosis, could protect against LPS-induced cardiac injury and also counteracted the deteriorating effects of TMEM43 silencing in the heart. Based on these findings, we concluded that TMEM43 protects against sepsis-induced cardiac injury via inhibiting ferroptosis in mice. By targeting ferroptosis in cardiomyocytes, TMEM43 may be a therapeutic strategy for preventing sepsis in the future.

## 1. Introduction

Worldwide, the incidence rate of sepsis-induced cardiomyopathy (SIC) varies from 10% to 70% [1]. Due to the lack of understanding of sepsis myocardial injury and the lack of unified diagnostic criteria, there are great differences in the diagnosis of sepsis myocardial injury [2]. However, once myocardial injury occurs in sepsis, it indicates high mortality and poor prognosis [2]. The mechanism of sepsis-induced cardiac dysfunction involves a variety of molecular pathways. The myocarditis cascade is driven by pathogen-associated molecular patterns (PAMPs) and damage-associated molecular patterns (DAMPs) [3]. Other mechanisms are also involved in mediating myocardial injury, including global myocardial ischemia, myocardial depressant substance, inflammation adrenergic pathways, calcium responsiveness, mitochondrial dysfunction, coronary microvascular dysfunction, and myocardial recovery [4]. The inherent complexity of pathophysiology involved in myocardial injury in sepsis and the variability between the patient and the organism cause a lot of difficulties in the treatment of myocardial injury [5]. Nevertheless, the exploration of pathophysiology of sepsis-induced cardiomyopathy will provide a basis for the treatment of SIC.

Iron is one of the essential trace elements for the human body. It is involved in the mitochondrial respiratory chain, nucleic acid replication, repair, and metabolism [6]. However, intracellular iron overload induces iron death and leads to organ dysfunction [7]. When glutathione (GSH) is consumed in vivo, the activities of GPx4 and iron death inhibitory protein 1 (FSP1) decrease, resulting in intracellular iron deposition, lipid peroxidation, and cell death [8]. Unlike apoptosis or necroptosis, iron death is characterized by inhibition by iron chelators and lipophilic antioxidants [9]. Studies have shown that iron death plays an important role in the occurrence of various cardiovascular diseases, induces cardiomyocyte death, and increases cardiac dysfunction [10]. Studies have shown that inhibition of ferroptosis-induced cardiomyocyte death can prevent myocardial ischemia–reperfusion injury [11]. GPx4 overexpression prevents [12] cardiomyocyte injury. Li et al. found that iron-dependent ferroptosis plays a key role in cardiovascular disease [13]. Ferroptosis inhibitor could reduce ferroptosis in myocardial cells and improve the cardiac function and survival rate of sepsis mice [13]. Therefore, the development of new treatments for ferroptosis may become a management and treatment strategy for sepsis.

Transmembrane protein 43 (TMEM43) is a four-transmembrane protein that is mainly localized in the endoplasmic reticulum and the inner nuclear membrane [14]. TMEM43 is a highly conserved protein expressed in most species [14]. A previous study found that TMEM43 mutation causes arrhythmogenic right ventricular dysplasia/cardiomyopathy [15]. Rouhi et al. also found that heterozygosity of TMEM43 in cardiomyocytes causes pro-fibrotic cardiomyopathy via activating the DNA damage response [16]. Systems genetics analysis revealed that TMEM43 was involved in cardiac- and metabolism-related pathways [17], suggesting that TMEM43 is closely associated with cardiovascular disease. However, whether TMEM43 is involved in the progression of SIC is unclear. In this study, we used an AAV9 delivery system to overexpress and knock down TMEM43, and found that TMEM43 protects against LPS-induced SIC via inhibiting ferroptosis. TMEM43 reduced the expression of P53, thus regulating the level of SLC7A11.

## 2. Materials and Methods

### 2.1. Animals and Animal Model

Beijing Union Medical College, Chinese Academy of Medical Sciences provided us with C57BL6 male mice (8–10 weeks old, weight 23.5–27.5g). Animal experiments were conducted in accordance with the guidelines for the care and use of laboratory animals (NIH Publication, revised 2011) published by the National Institutes of Health and approved by the Animal Use Committee of our hospital. Each mouse was subjected to LPS injection (L2630, Sigma, St. Louis, MO, USA) (the dose is 10 mg/kg, which is dissolved with normal saline for 12 h). Mice in the control group were injected with normal saline. One week before LPS injection, mice were subjected to injection of AAV9 harboring TMEM43 or shTMEM43 (each with 60μL, 5.0–6.5 × 1013 GC/mL) via retroorbital venous plexus. To determine the effect of ferroptosis on SIC, we treated mice with liproxstatin-1 (1 μM; Sigma-Aldrich) daily for 3 consecutive days before LPS injection. For the ferroptosis inhibitor, mice received intraperitoneal injection of Fer-1 (1 mg/kg) for 2 days, followed by LPS injection.

### 2.2. AAV9 Construction and Viral Delivery

Vigene Bioscience (Jinan, China) provided our designed AAV9-TMEM43 and AAV9-shTMEM43 (troponin T promoter was used to express in cardiomyocytes). Sixty to eighty microliters of AAV9- TMEM43 and AAV9-shTMEM43 at concentrations of 5.0–6.5 × 1013 GC/mL were used. Mice were injected with 60–80 μ L AAV9 (diluted with PBS solution and injected through retroorbital venous plexus), and LPS was injected one week later to establish the SIC model [18].

### 2.3. Echocardiography Measurements

MyLab 30CV (Esaote) echocardiography and a 15 MHz ultrasound probe were used for cardiac function detection. M-mode ultrasound and pulsed Doppler ultrasound were used. The left ventricular ejection fraction (LVEF) and left ventricular shortening fraction (LVSF) were calculated by measuring the size of the ventricular cavity and the thickness of the ventricular wall.

The mice were anesthetized with 1.5% isoflurane. After the feet of the mice did not respond to stimulation, left cardiac catheterization was used to measure the cardiac hemodynamic data of the mice. The catheter was inserted into the right carotid artery of the mouse and then sent to the left ventricle. The hemodynamic data of the left ventricle of the mouse during diastole and contraction were monitored using the Millar pressure volume system (mpvs-400; Millar instruments) (Powerlab/4SP A/D converter). PVAN software was used to process data.

### 2.4. Detection of Cardiac Injury Markers

Mice were injected with LPS, and the orbital venous blood was taken 12 h later. The serum was collected after 10 min of low-temperature high-speed centrifugation. Serum creatine kinase isozyme (CK-MB) was analyzed using an automatic biochemical analyzer (ADVIA ^®^ 2400, Siemens Ltd., Shanghai, China). Lactate dehydrogenase (LDH) was measured with an ELISA microplate reader (synergy HT, BioTek, Vermont, USA). The concentration of glutamic oxaloacetic transaminase (AST) was detected by an ELISA kit (Nanjing Jiancheng biological company, Nanjing, China).

### 2.5. Fe Assays and Malondialdehyde (MDA) Level

The levels of non-heme iron in serum and heart tissues were determined by an Fe detection kit (ab83366, Abcam). After fresh hearts were washed in precooled PBS, they were homogenized with Fe determination buffer on ice. The homogenate was collected and spun at 16,000 g for 10 min, and the supernatant was collected for use. The sample was diluted and 5 µL of Fe reducing agent was added. At the same time, different concentrations of standards were added and incubated at 37 °C for 30 min. One hundred microliters of Fe probe were incubated for 1h in the dark. The absorbance was read at 593 nm using a microplate reader. The Fe level was calculated according to the standard curve, and the protein concentration was used for normalization.

The MDA level was detected with a detection kit (Beyotime, bio., Shanghai, China). The absorbance was read at 532 nm, and the microplate reader was used.

### 2.6. Prussian Blue Staining

Heart slices were dewaxed at 60 °C, followed by hydration of the heart in distilled water. For the preparation of dyeing solution, equal volumes of potassium ferrocyanide solution and hydrochloric acid solution were mixed. Heart sections were incubated in staining solution for 3 min. Then, photos were taken with a light microscope (Olympic, Japan).

### 2.7. Cell Culture

H9c2 rat cardiomyocytes were purchased from the cell bank of the Chinese Academy of Sciences. Cells were cultured in DMEM containing 10% FBS (GIBCO). Cells were cultured in an incubator containing 5% CO_2_ at 37 °C (Sanyo 18m, Osaka, Japan). Cells were cultured with LPS (1 μg/mL) for 12 h. Cells were transfected with ad-TMEM43 or TMEM43 siRNA (purchased from Vigene Bioscience, China) to overexpress/silence TMEM43. Cells were cultured with erastin (10 μM, Medchemexpress, USA) or Fer-1 (0.5 μM, Medchemexpress, Monmouth Junction, NJ, USA) to induce/suppress iron death. Cells were treated with z-VAD-fmk (10  mM, Medchemexpress, USA) to inhibit apoptosis. Cell viability was detected by MTT assay (Beyotime, bio., Shanghai, China).

### 2.8. Detection of Reactive Oxygen Species (ROS)

The cells were digested into suspension cells with 0.25% trypsin. After centrifugation, the cells were resuspended with PBS. Cells were incubated with 2’, 7’-dichlorofluorescein diacetate (DCFH-DA) fluorescent probe for 20 min. Fluorescence intensity was detected by flow cytometry (Beckman, USA). Cell suspension was treated with 5 μ L c11-bodipy fluorescent probe was incubated to detect cellular lipid reactive oxygen species. The fluorescence intensity was detected by a microplate reader (synergy HT, BioTek, VT, USA) to calculate the cellular lipid reactive oxygen species.

Nicotinamide adenine dinucleotide phosphate (NADPH) oxidase activity was detected using a kit from beyotime (Shanghai, China).

### 2.9. RT-PCR and Western Blot

Total mRNA was isolated using TRIzol reagent. The mRNA purity was detected with a SmartSpec Plus spectrophotometer (bio RAD, Hercules, CA, USA) at (od260/od280 ratio). MRNA reverse transcription was performed using a cDNA synthesis kit (Roche Diagnostics). The LightCycler 480 SYBR Green I master kit was used for PCR amplification. GAPDH was used as the reference gene.

Primer used for TMEM43:
Mouse TMEM43
Sense: 5′-TGTACCAGTGGGTGGAGACA-3′
Antisense: 5′-AATGAGGCCTGCTGAGAGAA-3′
Rat TMEM43
Sense: 5′- TGTACCAGTGGGTGGAGACA-3′
Antisense: 5′- ACCTGCCAATCTGGACAAAG-3′

BCA method: after SDS-PAGE, the protein was transferred to polyvinylidene fluoride (PVDF) membrane (Millipore) followed by overnight incubation with primary antibodies. Primary antibodies included TMEM43, p53, SLC7A11, GPx4, ferritin, and GAPDH (purchased from Abcam) (1:100 dilution, Hercules, CA, USA). Blots were developed using enhanced chemiluminescence (ECL) reagents (bio RAD, Hercules, CA, USA). Image capture was performed by a ChemiDoc MP imaging system (bio RAD). GAPDH protein expression was used for normalization.

### 2.10. Data Analysis

Data are expressed as mean ± SD. SPSS 23.3 was used to analyze the data. A Student’s *t*-test was used to compare the two groups. One-way ANOVA and Tukey’s post hoc test were used to compare the data between the four groups. A *p*-value less than 0.05 was considered significant.

## 3. Results

### 3.1. The Expression Level of TMEM43 in Injured Hearts

**Figure 1:**
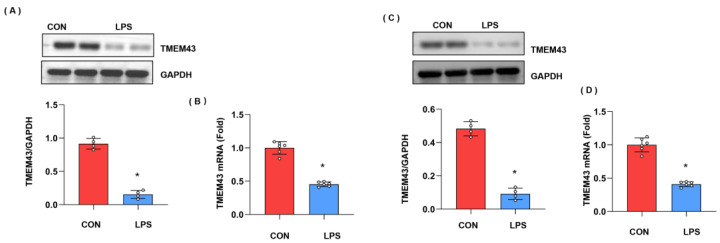
The expression level of TMEM43 in injured hearts. (**A**) Protein level of TMEM43 in mouse hearts challenged with LPS (*n* = 4). (**B**) mRNA level of TMEM43 in mouse hearts challenged with LPS (*n* = 6). (**C**) (*n* = 6). Protein level of TMEM43 in cardiomyocytes challenged with LPS (*n* = 4). (**D**) mRNA level of TMEM43 in cardiomyocytes challenged with LPS (*n* = 6). * *p* < 0.05 vs. CON group.

We first explore the expression level of TMEM43 during cardiac injury. As shown in Figure 1A,B, both the protein and mRNA expressions of TMEM43 were down-regulated after LPS stimulation. Moreover, we also assessed the level of TMEM43 in cardiomyocytes after LPS insult. As shown in Figure 1C,D, both the protein and mRNA level of TMEM43 was reduced in cardiomyocytes after LPS insult. These results suggest TMEM43 participates in the progression of cardiac injury.

### 3.2. Ferroptosis Plays an Essential Role in LPS-Induced Cardiac Injury

**Figure 2:**
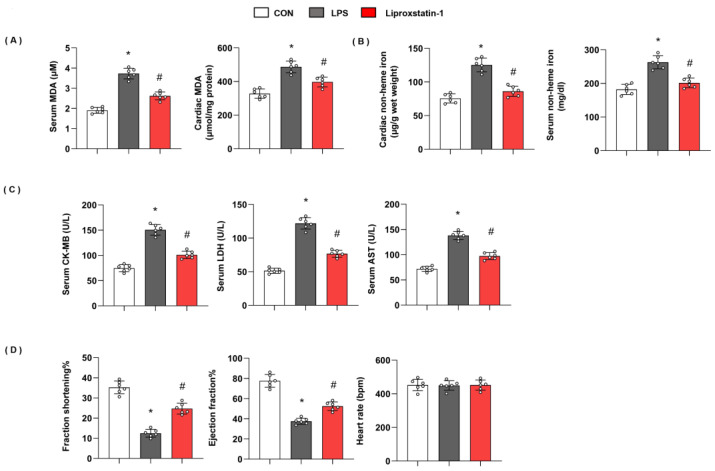
Ferroptosis plays an essential role in LPS-induced cardiac injury. (**A**) Serum and cardiac MDA level in mice injected with shTMEM43 under LPS challenge (*n* = 6). (**B**) Serum and cardiac non-heme iron in mice injected with shTMEM43 under LPS challenge (*n* = 6). (**C**) Serum CK-MB, LDH, and AST in each group (*n* = 6). (**D**) Echocardiography result in each group (*n* = 6). * *p* < 0.05 vs. CON group. # *p* < 0.05 vs. LPS group.

To confirm ferroptosis plays a deteriorating role in LPS-induced cardiac injury, we used liproxstatin-1 to inhibit ferroptosis. As shown in Figure 2, in mice with LPS injection, the level of serum MDA and cardiac MDA was raised, and non-heme iron in cardiac tissue and serum was also enhanced. However, serum MDA and cardiac MDA were reduced; non-heme iron in cardiac tissue and serum was also reduced in liproxstatin-1-treated mouse hearts (Figure 2A,B). Moreover, mice in the LPS group showed increased cardiac injury markers, including serum CK-MB, LDH, and AST, and deteriorating cardiac function. When we treated mice with liproxstatin-1, the cardiac injury markers reduced; cardiac function was also preserved (Figure 2C,D). These data indicated that ferroptosis plays an essential role in LPS-induced cardiac injury.

### 3.3. TMEM43 Knockdown Aggravates LPS-Induced Cardiac Injury

**Figure 3:**
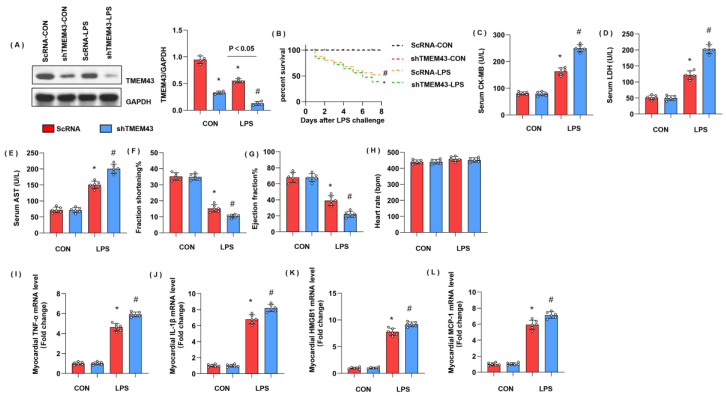
TMEM43 knockdown aggravates LPS-induced cardiac injury. (**A**) Protein level of TMEM43 in mouse hearts injected with shTMEM43 (*n* = 4). (**B**) Survival rate of mice in each group (*n* = 25). (**C**–**E**) Serum CK-MB, LDH, and AST in each group (*n* = 6). (**F**–**H**) Echocardiography result in each group (*n* = 6). (**I**–**L**) mRNA level of pro-inflammatory factors in heart tissue under LPS challenge (*n* = 6). * *p* < 0.05 vs. scRNA/CON group. # *p* < 0.05 vs. scRNA/LPS group.

To explore the role of TMEM43 in cardiac injury during sepsis, mice were subjected to AAV9-shTMEM43 to knock down TMEM43 and it was found that AAV9 delivery induced a sharp reduction in TMEM43 expression (Figure 3A). Mice in the LPS group showed an extremely high death rate and increased cardiac injury markers, including serum CK-MB, LDH, and AST. Meanwhile, mice in the AAV9-shTMEM43 group showed an aggravated death rate, and cardiac injury as assessed by enhanced level of serum CK-MB, LDH, and AST (Figure 3B–E). After LPS injection, mice in the LPS group revealed cardiac dysfunction with diminished LV fraction shortening and LV ejection fraction but preserved heart rate (Figure 3F–H). Mice in the AAV9-shTMEM43 group showed deteriorated cardiac dysfunction when compared with mice in the AAV9-scRNA group (Figure 3F–H). Cardiac inflammation response, the initial pathology of SIC, was also aggravated in the AAV9-shTMEM43 group, as evidenced by the increased mRNA level of tumor necrosis factor α (TNFα), interleukin-1 (IL-1), high mobility group protein 1 (HMGB1), and monocyte chemoattractant protein-1 (MCP-1) (Figure 3I–L).

### 3.4. TMEM43 Knockdown Aggravates LPS-Induced Ferroptosis

**Figure 4:**
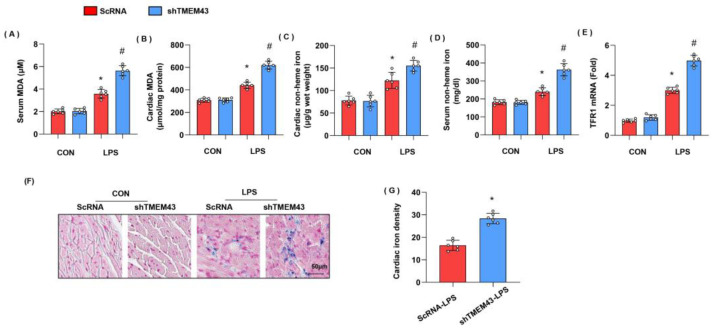
TMEM43 knockdown aggravates LPS-induced ferroptosis. (**A**,**B**) Serum and cardiac MDA level in mice injected with shTMEM43 under LPS challenge (*n* = 6). (**C**,**D**) Serum and cardiac non-heme iron in mice injected with shTMEM43 under LPS challenge (*n* = 6). (**E**) mRNA level of TFR1 in mouse hearts (*n* = 6). (**F**,**G**) Prussian blue staining and quantified results in mice injected with shTMEM43 under LPS challenge (*n* = 6). * *p* < 0.05 vs. scRNA/CON group. # *p* < 0.05 vs. scRNA/LPS group.

Previous studies have proved the essential role of ferroptosis in LPS-induced SIC [10]. To identify whether ferroptosis was activated, we detected the intermediate metabolites of lipid metabolism MDA, and found that both serum MDA and cardiac MDA level were remarkably increased in the LPS group when compared with that in the control group (Figure 4A,B). We also detected the levels of non-heme iron in cardiac tissue and serum as abnormal iron metabolism is the leading cause of ferroptosis. As a result, non-heme iron level was increased in both cardiac tissue and serum (Figure 4C,D). Circulating iron exists in the form of ferric iron (Fe^3+^) by binding to transferrin. Fe^3+^ is imported into cells through the membrane protein transferrin receptor 1 (TFR1). We detected the mRNA level of TFR1 and found that TFR1 was increased in LPS-treated mouse hearts, but enhanced in TMEM43 silenced mouse hearts (Figure 4E). Prussian blue staining showed that ferric iron was increased in the LPS group (Figure 4F). In mice with AAV9-shTMEM43 injection, the level of serum MDA and cardiac MDA was raised, non-heme iron in cardiac tissue and serum was also enhanced, and ferric iron was increased when compared with that in mice in the AAV9-scRNA group under LPS insult (Figure 4G). These data indicate TMEM43 knockdown aggravates LPS-induced ferroptosis.

### 3.5. TMEM43 Overexpression Alleviates LPS-Induced Cardiac Injury

**Figure 5:**
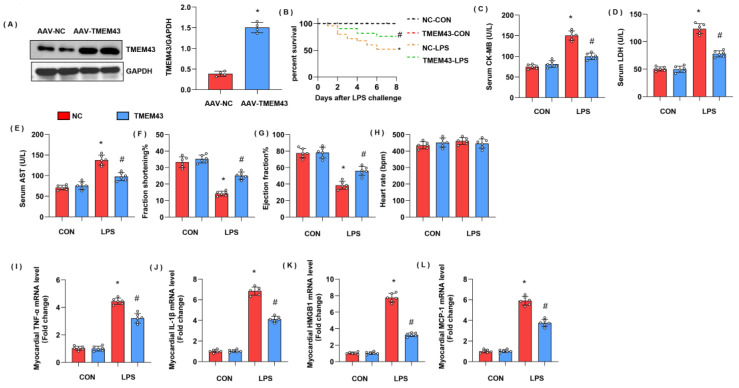
TMEM43 overexpression alleviates LPS-induced cardiac injury. (**A**) Protein level of TMEM43 in mouse hearts injected with AAV-TMEM43 (*n* = 4). (**B**). Survival rate of mice in each group (*n* = 25). (**C**–**E**) Serum CK-MB, LDH, and AST in each group (*n* = 6). (**F**–**H**) Echocardiography result in each group (*n* = 6). (**I**–**L**) mRNA level of pro-inflammatory factors in heart tissue under LPS challenge (*n* = 6). * *p* < 0.05 vs. AAV-NC/CON group. # *p* < 0.05 vs. AAV-NC/LPS group.

To explore whether overexpressed TMEM43 could alleviate cardiac injury during sepsis, mice were subjected to AAV9-TMEM43 to overexpress TMEM43 (Figure 5A). Mice in the LPS group showed an extremely high death rate and increased cardiac injury markers, including serum CK-MB, LDH, and AST. Meanwhile, mice in the AAV9-TMEM43 group showed ameliorated death rate and cardiac injury as assessed by enhanced level of serum CK-MB, LDH, and AST (Figure 5B–E). After LPS injection, mice in the LPS group revealed cardiac dysfunction with diminished LV fraction shortening and LV ejection fraction but preserved heart rate (Figure 5F–H). Mice in the AAV9-TMEM43 group showed improved cardiac dysfunction when compared with mice in the AAV9-NC group (Figure 5F–H). Cardiac inflammation response, the initial pathology of SIC, was also relieved in the AAV9-TMEM43 group, as evidenced by decreased mRNA level of TNFα, IL-1, HMGB1, and MCP-1 (Figure 5I–L).

### 3.6. TMEM43 Overexpression Alleviates LPS-Induced Ferroptosis

**Figure 6:**
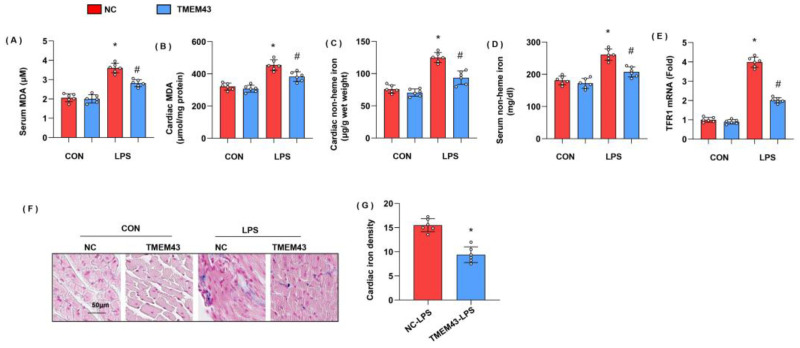
TMEM43 overexpression alleviates LPS-induced ferroptosis. (**A**,**B**) Serum and cardiac MDA level in mice injected with AAV-TMEM43 under LPS challenge (*n* = 6). (**C**,**D**) Serum and cardiac non-heme iron in mice injected with AAV-TMEM43 under LPS challenge (*n* = 6). (**E**) mRNA level of TFR1 in mouse hearts (*n* = 6). (**F**,**G**) Prussian blue staining and quantified results in mice injected with AAV-TMEM43 under LPS challenge (*n* = 6). * *p* < 0.05 vs. AAV-NC/CON group. # *p* < 0.05 vs. AAV-NC/LPS group.

To identify whether TMEM43 overexpression could suppress ferroptosis, we detected ferroptosis-associated evidence in AAV9-TMEM43-injected mice. Both serum MDA and cardiac MDA level were remarkably increased in the LPS group when compared with that in the control group (Figure 6A,B). Non-heme iron level was increased in both cardiac tissue and serum (Figure 6C,D). TFR1 was increased in LPS-treated mouse hearts, but reduced in overexpressed TMEM43 mouse hearts (Figure 6E). Prussian blue staining showed that ferric iron was increased in the LPS group (Figure 6F). In mice with AAV9-TMEM43 injection, the level of serum MDA and cardiac MDA dropped, non-heme iron in cardiac tissue and serum was also reduced, and ferric iron was diminished when compared with that in mice in the AAV9-NC group under LPS insult (Figure 6G). These data indicate TMEM43 overexpression could suppress LPS-induced ferroptosis.

### 3.7. TMEM43 Overexpression In Vitro Ameliorates LPS-Induced Cell Injury

**Figure 7:**
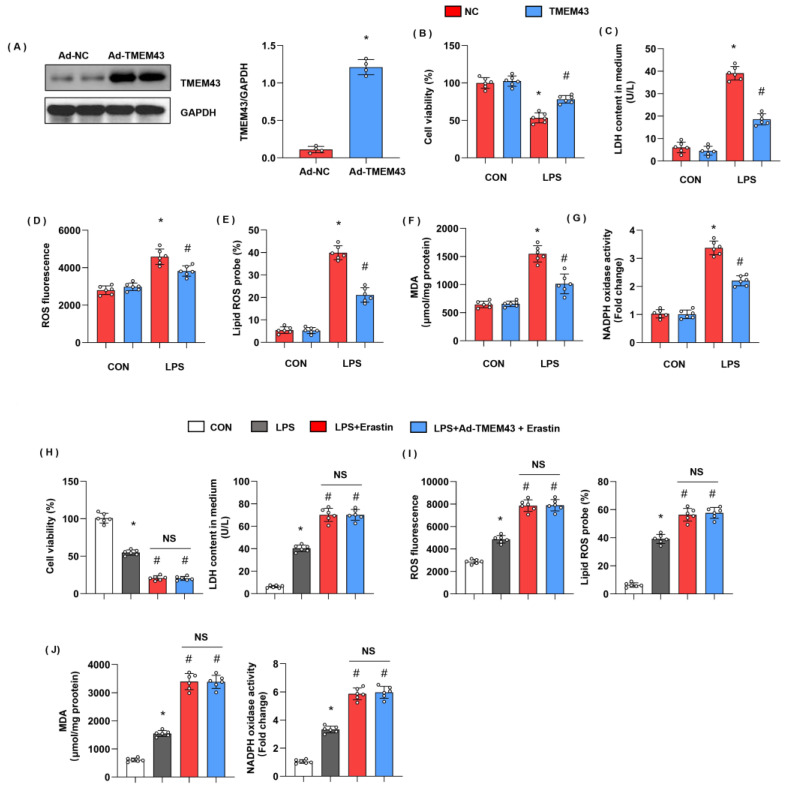
TMEM43 overexpression in vitro ameliorates LPS-induced cell injury. H9c2 cells were transfected with Ad-TMEM43. (**A**) Protein level of TMEM43 in H9c2 cells transfected with Ad-TMEM43 (*n* = 4). (**B**) Cell viability in each group (*n* = 6). (**C**) LDH level in H9c2 cells in each group (*n* = 6). (**D**) ROS level in H9c2 cells in each group (*n* = 6). (**E**) Lipid ROS level in H9c2 cells in each group (*n* = 6). (**F**,**G**) MDA and NADPH oxidase activity in each group (*n* = 6). * *p* < 0.05 vs. Ad-NC/CON group. # *p* < 0.05 vs. Ad-NC/LPS group. (**H**–**J**) H9c2 cells were transfected with Ad-TMEM43 and treated with erastin. (**H**) Cell viability and LDH level in H9c2 cells (*n* = 6). (**I**) ROS level and lipid ROS level in H9c2 cells in each group (*n* = 6). (**J**) MDA and NADPH oxidase activity in each group (*n* = 6). * *p* < 0.05 vs. CON group. # *p* < 0.05 vs. LPS group.

Meanwhile, we evaluated cell injury and ferroptosis in cardiomyocytes after LPS insult and explored the role of TMEM43 in cardiomyocytes. H9c2 cells were transfected with Ad-TMEM43 to overexpress TMEM43 (Figure 7A). After 12h of LPS stimulation, cell viability was reduced as compared with the control group (Figure 7B). LDH level was also raised in the LPS group (Figure 7C). The gross ROS level and the lipid ROS level were both increased after LPS challenge (Figure 7D,E). The MDA level and the NADPH oxidase activity were sharply enhanced after LPS challenge (Figure 7G). Meanwhile, cells in the TMEM43 overexpression group showed increased cell viability, decreased LDH level, gross ROS level, and lipid ROS level, and diminished MDA level and NADPH oxidase activity (Figure 7B–G). To confirm the effects of TMEM43 on ferroptosis, we used a ferroptosis inducer, erastin. As shown in Figure 7H–J, erastin deteriorated cell injury and oxidative injury when compared with the LPS group. Meanwhile, those injury parameters in cells treated with both Ad-TMEM43 and erastin showed no difference from those of cells in the erastin group.

### 3.8. TMEM43 Knockdown In Vitro Deteriorates LPS-Induced Cell Injury

**Figure 8:**
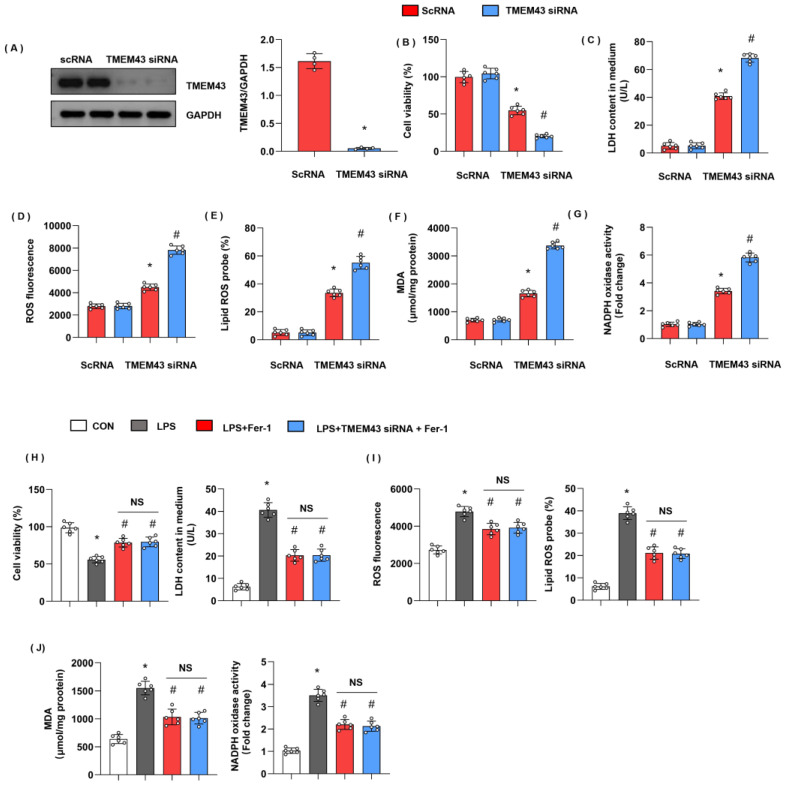
TMEM43 knockdown in vitro deteriorates LPS-induced cell injury. H9c2 cells were transfected with TMEM43 siRNA. (**A**) Protein level of TMEM43 in H9c2 cells transfected with TMEM43 siRNA (*n* = 4). (**B**) Cell viability in each group (*n* = 6). (**C**) LDH level in H9c2 cells in each group (*n* = 6). (**D**) ROS level in H9c2 cells in each group (*n* = 6). (**E**) Lipid ROS level in H9c2 cells in each group (*n* = 6). (**F**,**G**) MDA and NADPH oxidase activity in each group (*n* = 6). * *p* < 0.05 vs. scRNA/CON group. # *p* < 0.05 vs. scRNA/LPS group. (**H**–**J**) H9c2 cells were transfected with TMEM43 siRNA and treated with Fer-1. (**H**) Cell viability and LDH level in H9c2 cells (*n* = 6). (**I**) ROS level and lipid ROS level in H9c2 cells in each group (*n* = 6). (**J**) MDA and NADPH oxidase activity in each group (*n* = 6). * *p* < 0.05 vs. CON group. # *p* < 0.05 vs. LPS group.

We evaluated cell injury and ferroptosis in cardiomyocytes after LPS insult and explored the role of TMEM43 silencing in cardiomyocytes. H9c2 cells were transfected with TMEM43 siRNA to knock down TMEM43 (Figure 8A). After 12h of LPS stimulation, cell viability was reduced as compared with the control group (Figure 8B). LDH level was also raised in the LPS group (Figure 8C). The gross ROS level and the lipid ROS level were both increased after LPS challenge (Figure 8D,E). The MDA level and the NADPH oxidase activity were sharply enhanced after LPS challenge (Figure 8G). Meanwhile, cells in the TMEM43 silenced group showed decreased cell viability, enhanced LDH level, gross ROS level, and lipid ROS level, and increased MDA level and NADPH oxidase activity (Figure 8B–G). To confirm the effects of TMEM43 on ferroptosis, we used a ferroptosis inhibitor, Fer-1. As shown in Figure 8H–J, Fer-1 alleviated cell injury and oxidative injury when compared with the LPS group. Meanwhile, TMEM43 silencing could not change the protective effects of Fer-1 on cell injury.

### 3.9. TMEM43 Suppresses P53-Mediated Ferroptosis

**Figure 9:**
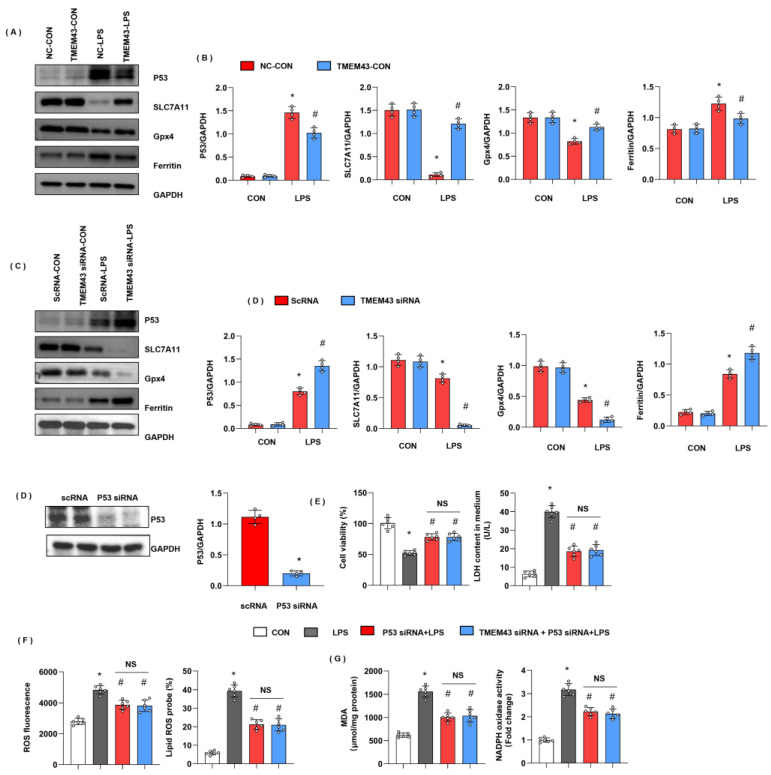
TMEM43 suppresses P53-mediated ferroptosis. (**A**,**B**) Protein level of P53, SLC7A11, Gpx4, ferritin in mouse hearts injected with AAV-TMEM43 under LPS challenge (*n* = 4). * *p* < 0.05 vs. AAV-NC/CON group. # *p* < 0.05 vs. AAV-NC/LPS group. (**C**,**D**) Protein level of P53, SLC7A11, Gpx4, ferritin in H9c2 cells transfected with TMEM43 siRNA under LPS challenge (*n* = 4). * *p* < 0.05 vs. scRNA/CON group. # *p* < 0.05 vs. scRNA/LPS group. (**E**–**G**) H9c2 cells were transfected with TMEM43 siRNA and P53 siRNA. (**E**) Cell viability and LDH level in H9c2 cells (*n* = 6). (**F**) ROS level and lipid ROS level in H9c2 cells in each group (*n* = 6). (**G**) MDA and NADPH oxidase activity in each group (*n* = 6). * *p* < 0.05 vs. CON group. # *p* < 0.05 vs. LPS group.

We assessed the ferroptosis-associated protein level. Ferritin is the main protein for intracellular iron storage, the abnormal level of which gives rise to iron metabolism disorder in cells. As the critical protein of ferroptosis, ferritin protein level was increased in the LPS group. Gpx4, a critical enzyme regulating lipid peroxidation, was decreased in the LPS group. SLC7A11, used for cysteine transport for GSH synthesis, was also reduced in the LPS group (Figure 9A,B). TMEM43 overexpression decreased the level of ferritin and increased the level of SLC7A11 and Gpx4. P53, upstream of ferroptosis and reported to regulate SLC7A11 and Gpx4 transcription, was also increased in the LPS group, while it dropped by TMEM43 overexpression (Figure 9A,B). To confirm TMEM43 regulating P53 in cardiomyocytes, we detected the P53-SLC7A11 pathway in cells. P53 and ferritin were increased; SLC7A11 and Gpx4 were decreased in TMEM43 knockdown cardiomyocytes after LPS insult (Figure 9C,D). To confirm the effects of TMEM43 on P53, cells were transfected with P53 siRNA to knock down P53 (Figure 9E). P53 silencing showed increased cell viability and reduced LDH level. Moreover, P53 silencing inhibited LPS-induced oxidative injury (Figure 9F,G). Meanwhile, TMEM43 silencing could not change the protective effects of P53 silencing on LPS-induced cell injury (Figure 9F,G).

### 3.10. Ferrostatin-1 Blurs TMEM43 Knockdown-Induced Aggravating Effects In Vivo

**Figure 10:**
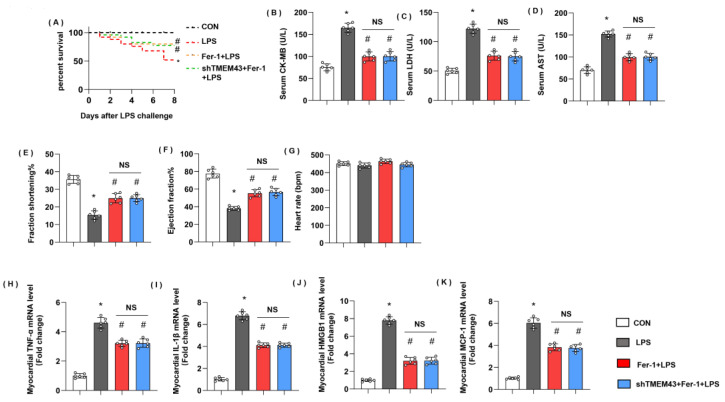
Ferrostatin-1 blurs TMEM43 knockdown-induced aggravating effects in vivo. Mice were subjected to shTMEM43 and Fer-1 as well as LPS challenge. (**A**) Survival rate of mice in each group (*n* = 25). (**B**–**D**) Serum CK-MB, LDH, and AST in each group (*n* = 6). (**E**–**G**) Echocardiography result in each group (*n* = 6). (**H**–**K**) mRNA level of pro-inflammatory factors in heart tissue under LPS challenge (*n* = 6). * *p* < 0.05 vs. CON group. # *p* < 0.05 vs. LPS group.

Next, we further identified the role of ferroptosis in TMEM’s deteriorating effects on SIC. Mice were treated with a specific ferroptosis inhibitor, Fer-1. Mice treated with Fer-1 showed ameliorated death rate and cardiac injury as assessed by reduced levels of serum CK-MB, LDH, and AST (Figure 10A–D). Mice in the Fer-1 group also revealed improved cardiac dysfunction with enhanced LV fraction shortening and LV ejection fraction (Figure 10E–H). Cardiac inflammation response, the initial pathology of SIC, was also alleviated in the Fer-1 group, as evidenced by decreased mRNA level of TNFα, IL-1, HMGB1, and MCP-1 (Figure 10H–K). However, in TMEM43 knockdown mice treated with Fer-1, the death rate and cardiac injury parameters showed no difference from the Fer-1 group. Taken together, ferroptosis maybe at least play part of the mechanism by which TMEM43 causes progression in SIC.

### 3.11. Apoptosis Inhibitor Could Not Blur the Protective Effects of TMEM43

**Figure 11:**
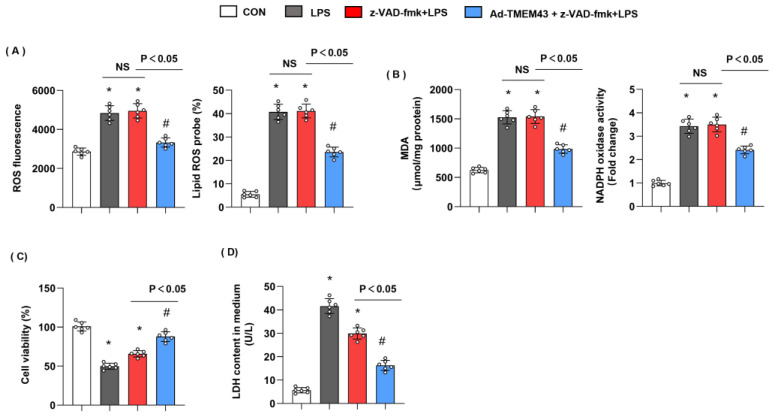
Apoptosis inhibitor could not blur the protective effects of TMEM43. (**A**) ROS level and lipid ROS level in H9c2 cells in each group (*n* = 6). (**B**) MDA and NADPH oxidase activity in each group (*n* = 6). (**C**,**D**) Cell viability and LDH level in H9c2 cells (*n* = 6). * *p* < 0.05 vs. CON group. # *p* < 0.05 vs. LPS group.

TMEM43 knockdown has previously been shown to promote apoptosis [16]. To rule out the effects of TMEM43 on apoptosis, cells were treated with apoptosis inhibitor z-VAD-fmk. As shown in Figure 11, LPS-induced oxidative injury was not hampered by z-VAD-fmk, while cells treated with both z-VAD-fmk and TMEM43 overexpression showed less oxidative injury. Cell viability was increased and LDH level was reduced in the z-VAD-fmk group, but TMEM43 overexpression further increased cell viability and reduced LDH level after LPS insult. Thus, our data suggest TMEM43 protects against SIC independent of apoptosis inhibitor.

## 4. Discussion

Severe sepsis can lead to septic cardiomyopathy, which is characterized by decreased cardiac ejection fraction, cardiomyocyte death, and interstitial edema [1]. At present, the diagnosis of septic cardiomyopathy includes echocardiography and detection of myocardial necrosis markers and inflammatory markers [2]. The pathogenesis of septic cardiomyopathy includes inflammatory storm, redox balance, myocardial cell injury, apoptosis, microvascular dysfunction, mitochondrial dysfunction, etc. [4]. According to the known case mechanism, vasoactive drugs, negative chronotropic drugs, anti-inflammatory drugs, and fluid resuscitation are used to treat septic cardiomyopathy [1]. However, once myocardial injury occurs, the prognosis of sepsis patients is poor [2]. In the present study, we found that TMEM43 was down-regulated in sepsis-induced cardiac injury heart tissue and cardiomyocytes. Decreased TMEM43 resulted in a lower death rate and less cardiac injury during sepsis. Decreased TMEM43 also caused ferroptosis, as characterized by increased ferric iron and lipid peroxidation. Subsequently, increased ferroptosis impaired cardiomyocyte contractility and even triggered cardiomyocyte death. Overexpression of TMEM43 sustained cardiac function and cardiomyocyte viability through controlled ferroptosis. Our study provides new insights into the molecular mechanism of the occurrence and development of septic cardiomyopathy. According to our results, drugs targeting the TMEM43–ferroptosis axis may benefit patients with septic cardiomyopathy.

Ferroptosis was discovered by Dr. Brent R. Stockwell in 2012. Iron death is obviously different from other types of cell death and is an Fe2+-reliant and ROS-dependent regulated cell death type [10]. Ferroptosis features changes in mitochondrial morphology and cristae structure and preserved integrity of the nucleus [13]. The mechanism of ferroptosis involves the formation of lipid pores. The accumulation of ferrous ions can lead to the oxidation of phospholipids containing polyunsaturated fatty acids (PUFAs), resulting in changes in the structure of cell membranes and increased membrane permeability, and ultimately leading to the rupture of cell plasma membranes and cell death [7,9]. Lipid peroxides, such as MDA, can inactivate cell structural proteins by crosslinking proteins, thereby promoting ferroptosis [19]. Ferroptosis is closely associated with various cardiovascular diseases, such as heart failure, myocardial ischemia/reperfusion injury, and SIC [10,20]. Inhibition of ferroptosis-induced cardiomyocyte death can prevent myocardial ischemia–reperfusion injury [21]. GPx4 overexpression prevents palmitic acid-induced ferroptosis and prevents cardiomyocyte injury [12]. Ferroptosis inhibitors could reduce ferroptosis in myocardial cells and improve the cardiac function and survival rate of sepsis mice [13]. In this study, we found that TMEM43 reduced the sepsis-induced ferroptosis as assessed by decreased ferric iron and lipid peroxidation. As a four-transmembrane protein anchored to the endoplasmic reticulum, a previous study found TMEM43 mutation causes arrhythmogenic right ventricular dysplasia/cardiomyopathy and TMEM43 was also reported to be associated with pro-fibrotic cardiomyopathy [15,16]. In this study, we first revealed the essential role of TMEM43 in SIC and its effects may regulate ferroptosis.

To evaluate the mechanism of TMEM43 in ferroptosis, we screened the associated signaling involved in ferroptosis and found that P53 was down-regulated by TMEM43.

Studies have found that P53 activation is necessary for cell ferroptosis in some cell types [22]. The abnormal metabolism of glutathione and GSH is the trigger factor of ferroptosis. GPx4 mainly scavenges lipid oxygen radicals [23,24]. The mechanism by which GPx4 inhibits ferroptosis is to reduce lipid hydroperoxides to non-toxic lipid alcohols [23]. One of the main components of GSH synthesis is cysteine, whose entry into the cell depends on system xc- [25]. The composition of heterodimeric solute carrier family 7 member 11 (SLC7A11) and solute carrier family 3 member 2 (SLC3A2) constitutes system xc-, which is used for cysteine transport [24]. Studies have shown that P53 can inhibit the transcription of SLC7A11, resulting in reduced cysteine transport, reduced GSH synthesis, and increased ferroptosis [26,27]. Rouhi et al. reported that haploinsufficiency of TMEM43 in myocytes caused impaired P53 inhibition, thus leading to DNA damage and pro-fibrotic cardiomyopathy [16]. Here, we found that TMEM43 could inhibit P53 expression, and subsequently activated SLC7A11 and Gpx4 to exert ferroptosis inhibition. When we used Fer-1, a ferroptosis inhibitor, the effects of TMEM43 silencing in hearts were blunted.

Ferritin is found in most tissues that store and keep iron in a soluble and non-toxic form [28]. It also represents an indirect marker of the total amount of intracellular iron, which increases with iron overload [28]. A previous study found that ferritin is decreased with sepsis-induced cardiac injury [13] and ferritin protects against ferroptosis [29]. However, in our study we found that ferritin was increased in SIC heart tissue and reduced by TMEM43. In an acute lung injury model, ferritin was reported to be increased and functioned as a negative factor to promote ferroptosis [28]. We take that increased ferritin may be a compensatory effect of iron overload in our model. TMEM43 reduced TFR1, thus suppressing iron import into cells. This may account for the reduced ferritin level with TMEM43. We notice that TMEM43 knockdown in mice did not show any difference in cardiac function under physical conditions. Since we knocked down the TMEM43 level in hearts by the AAV9 delivery system, the remaining expression of TMEM43 may cover the physical function of mouse hearts under normal conditions. Moreover, we only observed for 2 weeks after AAV9 injection, and the impact on cardiac function may occur at a later time. Thus, further study by using TMEM43 cardiac-specific knockout mice and a longer observation time is needed to gain knowledge of TMEM43 in the heart.

In summary, we found that TMEM43 decreased LPS-induced SIC and acted as a beneficial factor, which suppressed ferroptosis in cardiomyocytes and led to alleviated cardiac injury. Furthermore, we first observed that TMEM43 regulates ferroptosis in SIC via modulating the P53-SLC7A11 pathway.

## Data Availability

All the data will be available if required from the corresponding author.
